# Synthesis of Well-Defined Poly(*N*-H Benzamide-*co*-*N*-Octyl Benzamide)s and the Study of their Blends with Nylon 6

**DOI:** 10.3390/polym9050172

**Published:** 2017-05-13

**Authors:** Chih-Feng Huang, Miao-Jia Chen, Ching-Hsuan Lin, Yeo-Wan Chiang

**Affiliations:** 1 Department of Chemical Engineering, National Chung Hsing University, 250 Kuo Kuang Road, Taichung 402, Taiwan; g101065010@gmail.com (M.-J.C.); linch@dragon.nchu.edu.tw (C.-H.L.); 2 Department of Materials and Optoelectronic Science, Center for Nanoscience and Nanotechnology, National Sun Yat-Sen University, Kaohsiung 804, Taiwan; ywchiang@mail.nsysu.edu.tw

**Keywords:** chain-growth condensation polymerization, polybenzamide, Nylon 6, polymer blends

## Abstract

We synthesized a series of copolybenzamides (PBA) through chain-growth condensation polymerization (CGCP) of 4-(octylamino)benzoate (M4OB) and methyl 3-(4-(octyloxy)benzylamino) benzoate (M3OOB) co-monomers. Well-defined copolybenzamides with close to theoretical molecular weights (*M*_n_ ≈ 10,000–13,000) and narrow molecular weight distributions (*M*_w_/*M*_n_ < 1.40) were obtained. Selective removals of the protecting group (i.e., 4-(octyloxy)benzyl group) from the affording P(M3OOB-*co*-M4OB) copolybenzamides were subsequently performed to obtain P(M3NH-*co*-M4OB) copolymers. These novel *N*-H-containing copolybenzamides (named as PNHBA) can not only provide hydrogen bonds for polymer-polymer blends but also have good solubility in organic solvents. Miscibility of the PNHBA and Nylon 6 blends was investigated by differential scanning calorimetry (DSC), thermogravimetric analysis (TGA), FT-IR, contact angle analysis, transmission electron microscope (TEM), and dynamic mechanical analysis (DMA). This study illustrates a novel type of copolybenzamide with controlled molecular weight and narrow molecular weight distribution through an effective synthetic strategy, and can be applied to a practical blend of Nylon 6 with good miscibility.

## 1. Introduction

The development of high-performance polymers has been an important and long-term target of the past few decades. The study of polymer blends continues to draw attention from both a practical point of view and in fundamental research for the more precise understanding of the factors dominating polymer miscibility [1]. Moreover, the simple and effective polymer blending technique has generated new materials with combinations of the tailored properties that cannot be obtained in individual polymers. It is, thus, important to study the miscibility and phase behavior of polymer blends. Most polymer blends are immiscible/incompatible because of the unfavorable enthalpy and entropy of polymer-polymer mixing. The polymer miscibility can be enhanced through specific interactions, such as van der Waals forces, dipole-dipole interactions, hydrogen bonding, and electrostatic forces. The enthalpy and entropy changes associated with different types of hydrogen bonds have been investigated quantitatively from equilibrium constants using spectroscopy. These studies have illustrated that the contribution of the hydrogen bond to the free energy of mixing strongly relies on the amount and type of the hydrogen bonds in the mixture [2]. For example, the good miscibility of polyamides via hydrogen bonding depends on the balance of the negative contribution of their own molecular binding force among the original polyamide chains (i.e., self-association) and the positive contribution of their interaction with other polymer chains (i.e., inter-association) [3,4,5,6,7,8].

Polyamides have been mass-produced for decades. Among them, aliphatic polyamides, such as polyamide 6 (Nylon 6) and polyamide 66 (Nylon 66), are the most widely used in daily life. Recently, the high demand for smart/functional textiles has driven the investigation of novel polyamide blends to overcome the drawbacks of aliphatic polyamides, such as hydroscopicity and a narrow processing window. Regarding their chemical structures, blends of aliphatic and aromatic polyamides may provide a facile approach to enhance the property of aliphatic polyamides. Some studies have reported the presence of inter-association hydrogen bonding between aliphatic and aromatic polyamides resulted in good miscibility/compatibility [9,10,11,12,13,14,15,16,17,18]. In these studies, the entropy of mixing (Δ*S*_m_) is basically omitted and, thus, the free energy of mixing (Δ*G*_m_) can be regarded as the enthalpy of the mixing value (Δ*H*_m_) (i.e., Δ*G*_m_ ≒ Δ*H*_m_) which is represented as k*T*χ*ψ*_1_*ψ*_2_, where k = Boltzmann constant, *T* = absolute temperature, χ = interaction parameter, and *ψ_i_* = volume fraction of polymer *i* in the blending system [19]. These reports reveal the importance of inter-association hydrogen bonding in aliphatic and aromatic polyamide blends.

Since 2000, Yokozawa and coworkers have been developing chain-growth condensation polymerization (CGCP) to achieve the synthesis of condensation polymers by manipulating the substituent effects of monomers [20,21,22,23]. The reactions differ from conventional polycondensation reactions in that the monomers are highly selective in the reaction and react dominantly with the polymer end groups during CGCP, resulting in precise control of the molecular weight (*M*_W_), molecular weight distribution (MWD), and functionality. In the preparation of well-defined polybenzamides (PBAs) through CGCP (Scheme S1, see the Supplementary Material), deprotonation of the monomers using a strong base significantly deactivates the ester carbonyl group via a resonance/inductive effect to form meta-stable intermediate compounds and, therefore, the undesired reaction among the monomers is suppressed. After adding an initiator with a reactive ester carbonyl group, the reaction between the initiator and intermediates is highly favorable and a new amide linkage is formed. Simultaneously, the chain-ended ester group is re-activated from the weak electron-donating ability of the newly-formed amide linkage. Thus, the chemical structures of the initiators: *N*-substituents (i.e., the R^1^ groups), the leaving groups (i.e., the R^2^ groups), strong bases, and the polymerization temperatures can be altered to efficiently obtain homo-polybenzamides as well as block copolybenzamides [24,25,26,27], and star-shaped [28,29,30,31,32,33,34] and branched polybenzamides [35,36,37,38]. However, for preparing novel well-defined polybenzamides, the copolymerization of mixed monomers has not been explored to date.

**Scheme 1:**
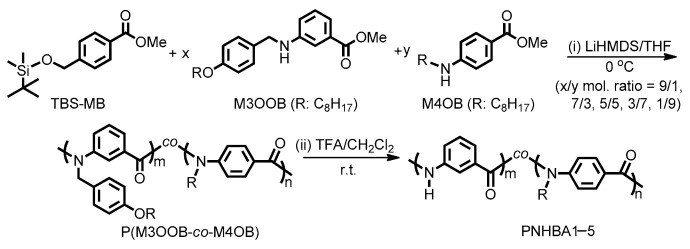
Synthesis of well-defined P(M3OOB-*co*-M4OB) copolybenzamides through CGCP of M3OOB and M4OB and selective deprotection of the pendent OOB group.

In this study, we synthesized a series of copolybenzamides through CGCP of 4-(octylamino)benzoate (M4OB) and methyl 3-(4-(octyloxy)benzylamino)benzoate (M3OOB) *co*-monomers (Scheme 1). Selective removal of the protecting group (i.e., 4-(octyloxy)benzyl (OOB) group) from five P(M3OOB-*co*-M4OB) copolybenzamides (PBA1–5) was subsequently performed to obtain P(M3NH-*co*-M4OB) copolymers (PNHBA1–5). We can expect that the PNHBAs not only provide hydrogen bonds but also have good solubility in organic solvents. Blends of PNHBA and Nylon 6 were further investigated by DSC, TGA, FT-IR, contact angle analysis, TEM, and DMA. Our aim was to demonstrate a novel type of polybenzamide obtained using an effective synthetic strategy to be applied to Nylon 6 blends.

## 2. Materials and Methods

### 2.1. Materials

Methyl 4-(*tert*-butyldimethylsiloxy)benzoate (TBS-MB), methyl 4-(octylamino)benzoate (M4OB) and methyl 3-(4-(octyloxy)benzylamino)benzoate (M3OOB) were synthesized according to previous studies [26,27,34]. One mole of lithium hexamethyldisilazide/tetrahydrofuran (1.0 M LiHMDS/THF solution), 1.0 M tetrabutylammonium fluoride/tetrahydrofuran (1.0 M TBAF/THF solution), LiCl (99%), NH_4_Cl (99.5%), and trifluoroacetic acid (TFA, 98%) were used as received from Sigma–Aldrich (St. Louis, MO, USA). Nylon 6 (NY6) was obtained from Du Pont (Santa Barbara, CA, USA) (Zytel 211^®^; *M*_n_ = 41,000 and *M*_w_/*M*_n_ = 2.93). All of the solvents were dehydrated prior to use.

### 2.2. Synthesis of P(M3OOB-co-M4OB) via CGCP

For detailed reaction procedures, the reader is referred to the previous studies [26,27,34]. A general example of PBA1: A mixture of 1.0 M LiHMDS/THF (5 mL, 5.0 mmol), LiCl (2.1 g, 50 mmol), initiator TBS-MB (84 mg, 0.25 mmol), M3OOB (3.35 g, 9.0 mmol), M4OB (0.21 g, 1.0 mmol), and 15 mL dry THF were charged into a 50 mL flask equipped with a three-way cock. The living polycondensations were conducted under nitrogen at −10 °C. All of the other copolymerizations (abbr.: PBA2–5) with different ratios of 70/30, 50/50, 30/70, and 10/90 were conducted in the same procedures. After the reaction finished, the mixture was quenched with saturated NH_4_Cl_(aq)_ and extracted with CH_2_Cl_2_ three times. The collected organic layer was washed with water and dried over MgSO_4_. The concentrated crude was re-dissolved in CH_2_Cl_2_ and reprecipitated into methanol/water = 9/1 (*v*/*v*) to afford a yellowish viscous P(M3OOB-*co*-M4OB) copolybenzamide (i.e., PBA1: *M*_n_ = 12,800, *M*_w_/*M*_n_ = 1.24, yield 85%). The reaction conditions and characteristics of the five polybenzamide copolymers (PBAs) were summarized in Table 1.

### 2.3. Selective Removal of 4-(octyloxy)benzyl Group (OOB) from PBAs 

An example: A 25 mL-flask equipped with a three-way stopcock was dried, purged with nitrogen, and PBA1 (200 mg) and dry CH_2_Cl_2_ (0.5 mL) were added. TFA (7 mL) was added and the mixture was stirred at room temperature for three days under nitrogen for the removal of the OOB group. The concentrated crude product was dissolved in CH_2_Cl_2_ and reprecipitated into hexane three times to afford PNHBA1 as a yellowish-white powder (72 mg, yield 78%).

### 2.4. Blend Preparation of PNHBA and NY6

PNHBA1 copolymer and Nylon 6 (NY6) with different weight ratios were dissolved in a co-solvent containing trifluoroethanol and chloroform (4:1 *v*/*v*) [9] with a solution concentration of ca. 3 wt % solid content. The mixture was precipitated into a non-solvent of methanol with vigorous stirring. The collected fine powder was washed extensively by methanol, repeatedly, and dried under vacuum for one day at 50 °C.

### 2.5. Characterization

Gel permeation chromatography (GPC) was equipped with: a Waters 515 pump (Hewlett Packard, Palo Alto, CA, USA), a Waters 410 differential refractometer (Hewlett Packard, Palo Alto, CA, USA), and two PSS SDV columns (PSS, Mainz, Germany) (Linear S and 100 Å pore size). The polymers were tested at 40 °C, USAing tetrahydrofuran (THF) with a flow rate of 1 mL/min. Polystyrene standards with different MWs and narrow MWDs were used for calibration. Proton nuclear magnetic resonance (^1^H NMR) analysis of the copolymers was performed using a Bruker 400 MHz system (Bruker, Billerica, MA, USA). The chemical shift was calibrated by setting the internal standard of deuterated DMSO at 2.49 ppm or CDCl_3_ at 7.26 ppm. Fourier transfer infrared (FT-IR) analysis of the blends was conducted using a Nicolet Avatar 320 FT-IR spectrometer (Nicolet, Madsion, WI, USA) under N_2_ to keep sample piece dry and to remove background noise. The polymer was mixed with a KBr pellet and pressed to form the sample for analysis. The glass transition (*T*_g_), crystallization (*T*_c_), and melting (*T*_m_) temperatures of the blends were characterized by a Seiko 6220 differential scanning calorimetry (DSC) (Seiko, Torrance, CA, USA) with a protocol as follows: (1) temperature was increased from 0 to 280 °C, and the sample was left for 30 min to remove thermal history, and then was quenched by liquid nitrogen; (2) the heat flow from −40 to 280 °C (with a ramp of 20 °C/min) was recorded under nitrogen. To perform thermogravimetric analysis (TGA) of the blends, a TA Instrument Q50 analyzer (scan rate = 20 °C/min from 30 to 700 °C under nitrogen) with a platinum holder was used. The *T*_d5%_ values were determined from the 5% decomposition of the samples. The water contact angle (CA) of the blends was characterized by the KRÜSS G10 system (KRÜSS, Hamburg, Germany) under ambient conditions. Solvent casting films were stained by RuO_4_ and the microstructure images were detected by a JEOL JEM-2100 transmission electron microscope (TEM) (JEOL, Tokyo, Japan). For dynamic mechanical analysis (DMA), samples were prepared by solvent casting to a dimension of 0.05 × 10 × 30 mm^3^. The analysis was recorded by a PerkinElmer DMA 8000 (PerkinElmer, Waltham, MA, USA) from 20 to 180 °C (with a ramp of 20 °C/min) under nitrogen in tensile mode.

## 3. Results and Discussion

**Figure 1:**
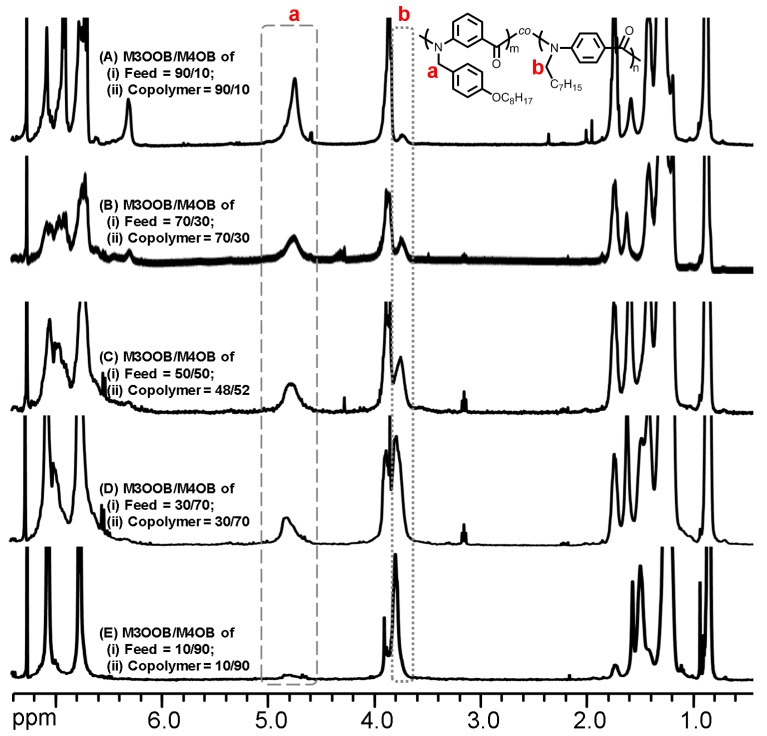
^1^H NMR spectra (400 MHz, CDCl_3_) of P(M3OOB-*co*-M4OB) with different molar feed ratios of M3OOB/M4OB: (**A**) 90/10; (**B**) 70/30; (**C**) 50/50; (**D**) 30/70; and (**E**) 10/90 ((ii) values were determined by the ratios of the dash-region of *a* for characteristics of the M3OOB unit and the dot-region of *b* for characteristics of the M4OB unit).

Copolymerization of M3OOB and M4OB with different molar feed ratios was firstly carried out to examine the reactivity between the two monomers. The resulting copolymers were analyzed by ^1^H NMR. Figure 1 reveals the main assignments and compositions of P(M3OOB-*co*-M4OB) based on the peaks at 4.73 ppm (i.e., peak *a* from the M3OOB unit) and 3.79 ppm (i.e., peak *b* from the M4OB unit), respectively. Based on the ratio of the two peaks (i.e., *I*_a_/*I*_b_), the copolymerized fraction of M3OOB (*f*_1_) was estimated. The detailed characteristics of the copolymers were analyzed by GPC and are summarized in Table 1 and Table S1 (see Supplementary Materials). From the ^1^H NMR and GPC analysis, a significant relationship between the monomer feeds and copolymer compositions was observed and well-defined copolybenzamides with MWs close to theoretical values (*M*_n_ ≈ 10,000–13,000) and narrow MWDs (*M*_w_/*M*_n_ < 1.40) were shown to be obtained.

We further estimated the reactivity ratio of *r*_1(M3OOB)_ and *r*_2(M4OB)_ based on the molar ratios between the monomer feeds and copolymers by employing the Kelen–Tüdos method [8] as follows:

$$\eta =(r_{1}+\frac{r_{2}}{\alpha })\xi -\frac{r_{2}}{\alpha }\;\mathrm{where}\;\eta =G/(\alpha +H)\;\mathrm{and}\;\eta =H/(\alpha +H)$$

**Figure 2:**
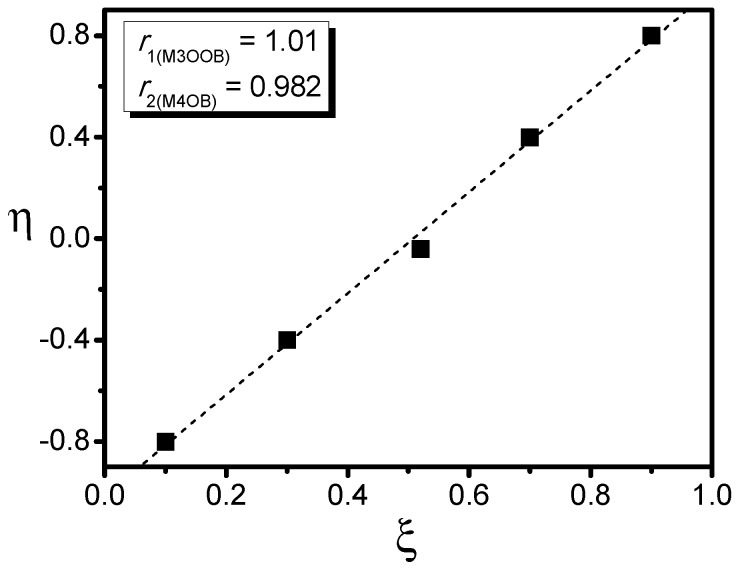
Kelen-Tüdos plot of M3OOB and M4OB copolymerizations.

*H* and *G* can be estimated using *H* = *x*^2^/*y* and *G* = *x*(*y* − 1)/*y*, where *x* = *F*_1_/*F*_2_ (i.e., the ratio of molar concentration of monomer 1 and 2 in the feed) and *y* = *f*_1_/*f*_2_ (i.e., the mole ratio of these monomers in the copolymer). *α* = √(*H*_m_*H*_M_) where *H*_m_ and *H*_M_ are the lowest and highest *H* values from the experiments. Figure 2 displays the Kelen–Tüdos plot of P(M3OOB-*co*-M4OB). By interpolating the experimental results (*η* as a function of *ξ*), we found *r*_1(M3OOB)_ = 1.01 and *r*_2(M4OB)_ = 0.982. The similar reactivity ratios indicate the ideal copolymerization behavior of the system. The sequence distribution of the copolymers was further estimated from the reactivity ratios based on the statistically conditional probabilities (*P*_ij_, as summarized in Table 1) [39,40]. It also illustrated that the copolymerization systems resulted in an ideal random distribution of the monomeric units in the copolymer chain. From results (i.e., *M*_n_ ≈ *M*_n,th_, *M*_w_/*M*_n_ < 1.4, *F*_1_ ≈ *f*_1_, the values of *P*_12_ and *P*_21_ and thermal properties (discussed later)), it is clear that well-defined copolybenzamides were obtained successfully through the proposed living “chain-growth” fashion, as shown in Scheme S1 (see Supplementary Materials).

**Figure 3:**
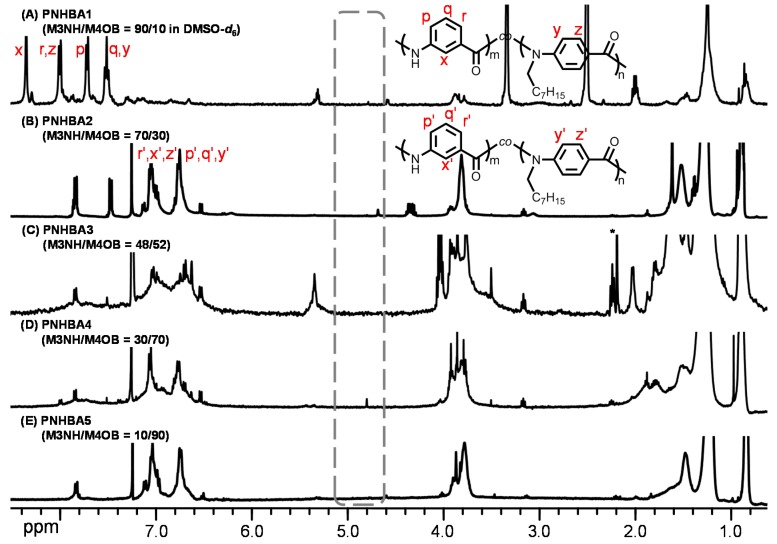
^1^H NMR spectra (400 MHz) of P(M3NH-*co*-M4OB) copolymers (**A**: in DMSO-*d*_6_, **B**–**E**: in CDCl_3_; PNHBA1–5 correspond to the samples in Figure 1, respectively; *: impurities).

Removal of the OOB protecting group in the P(M3OOB-*co*-M4OB) copolymers was further conducted to produce the P(M3NH-*co*-M4OB) copolymers, which possessed not only strong hydrogen bonding but also had good solubility in polar solvents. To facilitate the removal of the OOB group, trifluoroacetic acid (TFA) was utilized. Figure 3 shows the ^1^H NMR spectra (400 MHz, (A) in DMSO-*d*_6_; (B–E) in CDCl_3_) after treatment with TFA. Characteristic peaks in Figure 3a were further assigned to identify the chemical structure. The disappearance of the representative signals of the OOB group (e.g., *δ* (ppm) = 4.25–4.85) were clearly observed in the resulting copolymers of PNHBA1–5. The composition differences of the PNHBA1–5 samples are corresponding to the samples in Figure 1, respectively. Furthermore, the representative FT-IR spectra before and after treatment by TFA demonstrated that the amine group appeared and the amide linkages remained (see the Supplementary Materials, Figure S2). From these results, successful deprotection and good solvent solubility of the copolybenzamides were achieved.

**Figure 4:**
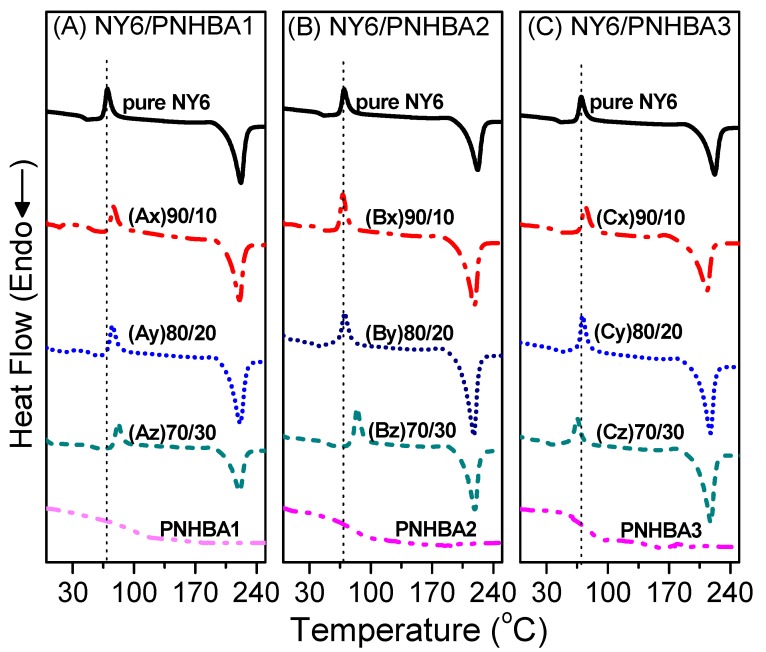
DSC traces of (**A**–**C**) NY6/PNHBA1–3 blends with different weight ratios (*x* = 90/10, *y*: 80/20 and *z*: 70/30).

**Figure 5:**
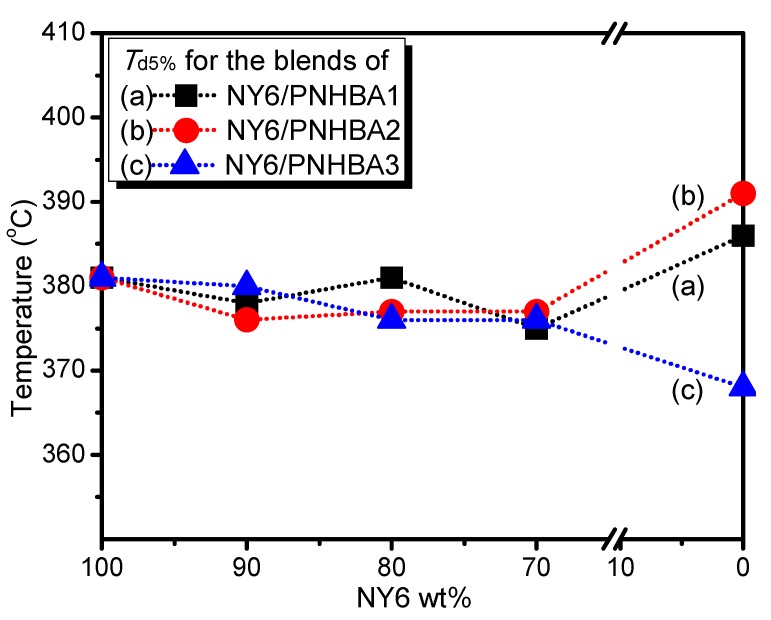
Summary of *T*_d5%_s of NY6/PNHBA1–3 blends with different weight percentages (**a**: NY6/PNHBA1 blends, **b**: NY6/PNHBA2 blends, and **c**: NY6/PNHBA3 blends).

In an attempt to investigate the compatibility of the copolymers, we blended the resulting PNHBA1–3 with Nylon 6 (NY6). Figure 4 shows the DSC traces of the three blended sets and the related characterization data are summarized in Table 2. Structurally speaking, highly rigid, symmetrical, and easily packed polymer chains result in high *T*_g_ and *T*_m_ properties. Furthermore, the more depressions in the *T*_g_ and *T*_m_ data, the higher the polymer-polymer miscibility or compatibility. However, it was observed that the melting behaviors of NY6 and NY6 in the presence of the miscible polymers were similar. We further estimated the variations between *T*_c_ and *T*_g_ (i.e., Δ*T*_cg_ = *T*_c_ − *T*_g_) to investigate the homogeneity of the blends [41,42,43]. In the NY6/PNHBA1 blends (Figure 4A), a clear increase in the *T*_g_ and the *T*_c_ was seen but little variation in *T*_m_ relative to NY6. With 30 wt% of PNHBA1, a low *X*_c_ (i.e., crystallinity) was acquired, which might be ascribed to suppression of the NY6 chain mobility because of the good homogeneity of the blends. In Figure 4B, where NY6/PNHBA2 = 70:30, a significant increase of approximately 14 °C in *T*_c_ and a 34 °C difference in Δ*T*_cg_ was observed relative to NY6. In the NY6/PNHBA3 blends (Figure 4C), a decrease in *T*_g_ was observed but *X*_c_ remained constant, which might be ascribed to a higher content of flexible segments (i.e., M4OB unit) in PNHBA3. Thus, the crystallinity of NY6 composites can be tuned from *ca.* 6% to 17% by varying the contents and compositions of the copolybenzamides. From the results, we can rationally deduce good miscibility among the NY6/PNHBAs blends. The thermal stability of the homo/copolymers and blends were then examined using TGA. As shown in Figure S3 (see Supplementary Materials), simple and smooth TGA curves were observed. For the copolybenzamides, the char yields were *ca.* 30% at 650 °C because of the high content of aromatic moiety. Accordingly, the char yields increased to ~10% with an increase in the amount of copolybenzamides. Figure 5 summarizes the *T*_d5%_ values from the TGA traces. In all blends, *T*_d5%_s were in the range of 375–380 °C, which indicates high thermal stability. These results imply that moderately compatible blending systems with good thermal stability were acquired.

**Figure 6:**
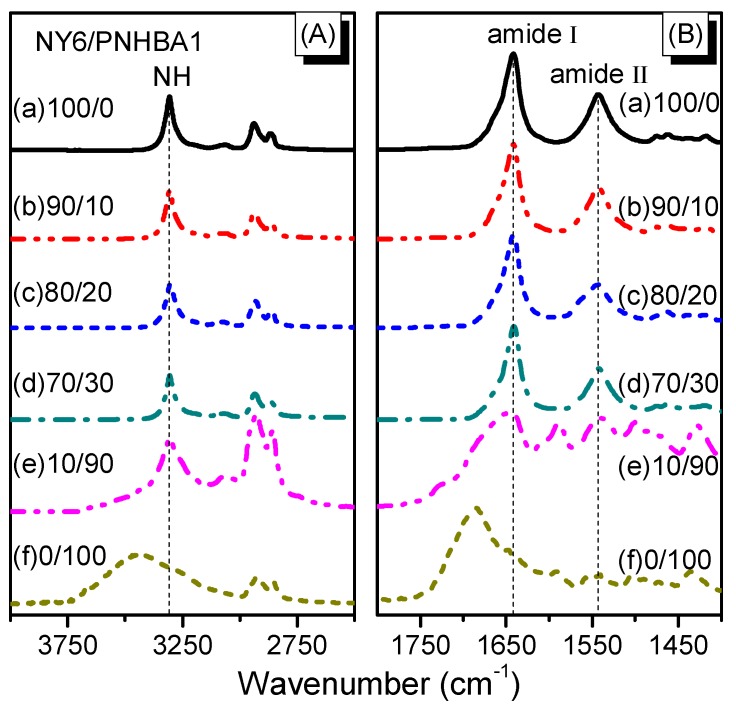
FT-IR spectra of NY6/PNHBA1 blends with different weight ratios measured at 100 °C in regions of (**A**) 4000–2500 and (**B**) 1800–1400 cm^−1^.

The inter-association interactions of the NY6/PNHBA1 blends were further analyzed by FT-IR at 100 °C. Figure 6 shows two regions of the FT-IR spectra ((A) 4000–2500 and (B) 1800–1400 cm^−1^) with various blending ratios. With a higher content of PNHBA1, broadening of the amine and amide I/II peaks (i.e., at 3300 and 1640/1540 cm^−1^) occurred, especially for the amide II peak (variations of half-height width: (a) 27, (b) 31, (c) 36, (d) 36, and (e) 39 cm^−1^). These results indicated moderate intermolecular hydrogen bonding between NY6 and PNHBA1 at 100 °C.

**Figure 7:**
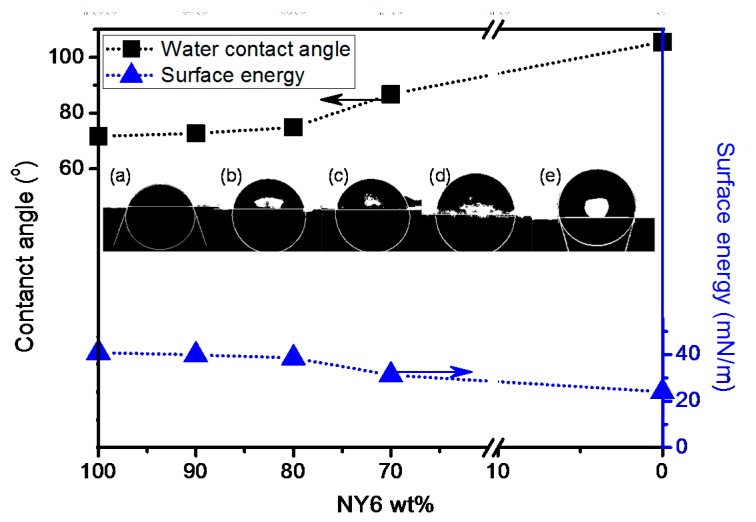
Surface hydrophilicity and energy of NY6/PNHBA1 blends (ratio of **a** = 100/0, **b** = 90/10, **c** = 80/20, **d** = 70/30, and **e** = 0/100).

As mentioned above, the incorporation of hydrogen bonding unit into the copolybenzamides indeed enhanced the compatibility between the NY6 and polybenzamide. Another benefit of our designed copolybenzamides is that the presence of alkyl side chain should enhance hydrophobicity of the composites with respect to the nature of high hydrophilicity of NY6. We, thus, examine the contact angle (CA) and surface energy (SE) of the NY6/PHNBA1 composites. As shown in Figure 7, the CAs/SEs of pure NY6 and pure PNHBA1 are ca. 71.7°/41.2 mN/m and 103.4°/24.0 mN/m, respectively. With increases of the blending amount of PNHBA1 (i.e., 90/10, 80/20, and 70/30), accordingly, the CAs and SEs gradually shifted toward the value of pure PNHBA1. This indicated an additivity effect of NY6/PNHBA1 blends.

**Figure 8:**
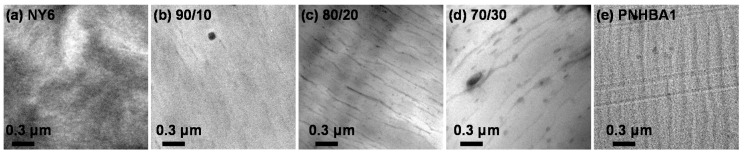
TEM images of NY6/PNHBA1 blends with different weight ratios (ratio of a = 100/0, b = 90/10, c = 80/20, d = 70/30, and e = 0/100; samples were stained by RuO_4_ excepting for pure NY6).

**Figure 9:**
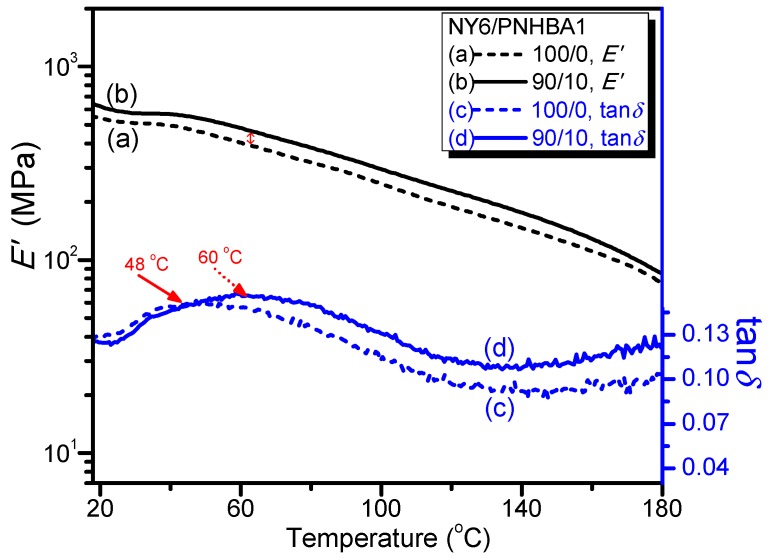
(**a**,**b**) Storage modulus (*E*’) and (**c**,**d**) tan *δ* of pure NY6 and NY6/PNHBA1 (9:1 *w*/*w*) blends measured by DMA.

We used TEM to visualize the homogeneity of the NY6/PNHBA1 blends. As shown in Figure 8, with the exception of pure NY6, all of the samples were stained by RuO_4_ to enhance the image contrast. The pure NY6 and PNHBA1 samples were homogeneous as shown in Figure 8a,e. For the blends (i.e., Figure 8b–d), deep-gray areas were observed, which are ascribed to the stainable parts of PNHBA1. The deep-gray stripes in Figure 8c,d are ascribed to the shear force while preparing the specimen by microtone. With an increase in the blending amount of PNHBA1, the deep-gray dots did not show significant aggregation. The deep-gray dots had an average diameter of 30–50 nm, which implies the homogeneous dispersion of PNHBA1 in NY6. This supports the observations in the DSC traces. In addition, we examined the mechanical property of the NY6/PNHBA1 sample (90:10 *w*/*w*) using DMA. Figure 9 demonstrates the storage modulus (*E*′) and tan *δ* of pure NY6 (dash curves) and the NY6/PNHBA1 sample (solid curves) in the temperature range between −40 and 180 °C. The presence of PNHBA1 increased the *E*′ property (i.e., curve a vs. b). In comparison to pure NY6, tan *δ* and *T*_g_ (~60 °C) increased with the presence of PNHBA1 (i.e., curve c vs. d). These observations are consistent with previous characterization results.

## 4. Conclusions

Using CGCP of M3OOB and M4OB co-monomers, we synthesized copolybenzamides with different compositions. A series of well-defined copolybenzamides with close to theoretical MWs (*M*_n_ ≈ 10,000–13,000) and narrow MWDs (*M*_w_/*M*_n_ < 1.40) were obtained. Using the Kelen-Tüdos method to estimate the monomer reactivity, similar reactivity ratios for the individual monomers (*r*_1(M3OOB)_ = 1.01 and *r*_2(M4OB)_ = 0.982) were acquired, indicating an ideal copolymerization behavior of the M3OOB and M4OB pair. Characterization by ^1^H NMR and FT-IR showed that selective removal of the OOB protecting group in the P(M3OOB-*co*-M4OB) copolymers was successfully achieved to form P(M3NH-*co*-M4OB) copolymers (abbr.: PNHBA). DSC traces of NY6/PNHBAs blends indicated that the crystallinity of NY6 composites could be altered from ca. 6% to 17% by varying the contents and compositions of the copolybenzamides, indicating good miscibility among the NY6/PNHBAs blends. From TGA curves, *T*_d5%_s of the blends were in the range of 375–380 °C, depicting their high thermal stability. We further performed FT-IR and water CA measurements to analyze the NY6/PNHBA1 blends, in which moderate inter-association interactions and an additivity effect was observed. In addition, from TEM and DMA measurements of the NY6/PNHBA1 blends, good homogeneity and enhancement of *E*’ and tan *δ* were seen, respectively. In summary, we demonstrated an effective strategy to synthesize a novel type of polybenzamide, which not only provides hydrogen bonding but also shows high solubility in organic solvents and good miscibility with Nylon 6.

## Figures and Tables

**Table 1:** Characteristics of the polybenzamide copolymers (PBAs).

Samples	*F*_1_^a^ (Feeds)	*f*_1_^a^ (Copolymers)	*M*_n,th_^b^	*M*_n,GPC_^c^	*M*_w_/*M*_n_	*P*_12_^d^	*P*_21_^d^
PBA1	0.90	0.90	13,330	12,800	1.24	0.099	0.902
PBA2	0.70	0.70	12,490	10,700	1.18	0.298	0.704
PBA3	0.50	0.48	11,640	9700	1.39	0.498	0.505
PBA4	0.30	0.30	10,790	11,300	1.29	0.698	0.304
PBA5	0.10	0.10	9940	10,400	1.06	0.899	0.102

^a^ Molar fractions of M3OOB/(M3OOB + M4OB) of feeds (F) and copolymers (*f* measured by ^1^H NMR); ^b^ Theoretical MWs were estimated based on the quantitative monomer consumptions plus MW of the initiator (i.e., *M*_n,th_ = *F*_1_ × MW_(M3OOB repeating unit)_ + *F*_2_ × MW_(M4OB repeating unit)_ + MW_initiator_); ^c^ MWs were estimated by GPC (eluent: THF) calibrated by polystyrene standards; ^d^ *P*_ij_: Conditional probabilities of the addition of monomer i to a growing chain-end with an active j radical. *P*_12_ = 1 − *P*_11_ = 1/(1 + *r*_1_*X*) and *P*_21_ = 1 − *P*_22_ = 1/(1 + *r*_2_*X*), where *X* = [M3OOB]_0_/[M4OB]_0_ [39,40].

**Table 2:** Summary of the DSC results of NY6/PNHBA1–3 blends.

Samples (No.)	*T*_g_ (°C)	*T*_c_ (°C)	Δ*T*_cg_^a^ (°C)	*T*_m_ (°C)	Δ*H*_m_ – Δ*H*_c_ (J/g)	*X*_c_ (%) ^b^
pure NY6	44.3	69.7	25.4	220.0	26.1	11.3
NY6/PNHBA1	-	-	-	-	-	-
90/10 (Ax)	50.0	76.4	26.5	220.7	22.42	9.7
80/20 (Ay)	49.3	75.2	25.9	221.7	23.14	10.1
70/30 (Az)	51.8	81.6	29.8	222.6	13.52	5.9
PNHBA1	99.0	-	-	-	-	-
NY6/PNHBA2	-	-	-	-	-	-
90/10 (Bx)	41.9	68.2	26.3	218.2	34.71	15.1
80/20 (By)	42.5	70.3	27.8	218.0	20.30	8.8
70/30 (Bz)	50.4	84.4	34.0	218.9	19.25	8.4
PNHBA2	79.9	-	-	-	-	-
NY6/PNHBA3	-	-	-	-	-	-
90/10 (Cx)	40.0	74.0	34.1	213.0	32.40	14.1
80/20 (Cy)	42.6	71.3	28.7	217.4	37.95	16.7
70/30 (Cz)	32.2	64.8	32.6	217.0	31.96	13.9
PNHBA3	73.5	-	-	-	-	-

^a^ Δ*T*_cg_ = *T*_c_ − *T*_g_; ^b^ Degree of crystallinity (*X*_c_) % = (Δ*H*_m_ – Δ*H*_c_)/Δ*H*_m_^o^ × 100% (*H*: enthalpy).

## Supplementary Materials

The following are available online at www.mdpi.com/2073-4360/9/5/172/s1, Scheme S1: CGCP mechanism for the synthesis of well-defined polybenzamides, Figure S1: ^1^H NMR spectra for the (A) initiator 4-(((*tert*-butyldimethyl silyl)oxy)methyl)benzoate; (B) monomer methyl 4-(octylamino)benzoate (M4OB), and (C) methyl 3-(4-(octyloxy)benzylamino)benzoate (M3OOB), Figure S2: Representative FT-IR spectra of (a) P(M3OOB-*co*-M4OB) and (b) the resulting PNHBA after deprotection of the OOB group, Figure S3: TGA traces and *T*_d5%_s of (a–c) NY6/PNHBA1–3 blends with different weight ratios, Table S1. Characteristics of polybenzamide copolymers (PBAs).

## References

[1] Krausch G. (1995). Surface-induced self-assembly in thin polymer-films. Mater. Sci. Eng. R.

[2] Coleman M.M., Graf J.F., Painter P.C. (1991). Specific Interactions and the Miscibility of Polymer Blends.

[3] Hu J.B., Painter P.C., Coleman M.M., Krizan T.D. (1990). Toward a prediction of the phase-behavior of polyamide blends. J. Polym. Sci. Part B Polym. Phys..

[4] Coleman M.M., Skrovanek D.J., Painter P.C. (1986). Hydrogen-bonding in polymers. 3. Further infrared temperature studies of polyamides. Makromol. Chem. Macromol. Symp..

[5] Wang C.-F., Su Y.-C., Kuo S.-W., Huang C.-F., Sheen Y.-C., Chang F.-C. (2006). Low-surface-free-energy materials based on polybenzoxazines. Angew. Chem. Int. Ed..

[6] Huang C.-F., Kuo S.-W., Lin F.-J., Huang W.-J., Wang C.-F., Chen W.-Y., Chang F.-C. (2006). Influence of PMMA-chain-end tethered polyhedral oligomeric silsesquioxanes on the miscibility and specific interaction with phenolic blends. Macromolecules.

[7] Huang C.-F., Kuo S.-W., Lin F.-J., Wang C.-F., Hung C.-J., Chang F.-C. (2006). Syntheses and specific interactions of poly(hydroxyethyl methacrylate-*b*-vinyl pyrrolidone) diblock copolymers and comparisons with their corresponding miscible blend systems. Polymer.

[8] Huang C.-F., Chang F.-C. (2003). Comparison of hydrogen bonding interaction between PMMA/PMAA blends and PMMA-*co*-PMAA copolymers. Polymer.

[9] Ellis T.S. (1988). Miscibility in blends of aliphatic polyamides and an aromatic polyamide, Nylon 3Me6T. Polymer.

[10] Ellis T.S. (1989). Miscibility and immiscibility of polyamide blends. Macromolecules.

[11] Ellis T.S. (1990). Aromatic polyamide blends—Enthalpy relaxation and its correlation with phase phenomena. Macromolecules.

[12] Ellis T.S. (1990). Critical miscibility limits in blends of aliphatic polyamides containing an aromatic polyamide. Polymer.

[13] Ellis T.S. (1990). The role of repulsive interactions in polyamide blends. Polym. Eng. Sci..

[14] Ellis T.S. (1991). Influence of structure on phase-behavior of polyamide blends. Macromolecules.

[15] Ellis T.S. (1992). Mixing relationships in aliphatic polyamide blends. Polymer.

[16] Ellis T.S. (1993). Polyamide polyester blends—An estimation of the amide-ester interaction. J. Polym. Sci. Part B Polym. Phys..

[17] Ellis T.S. (1996). Estimating interactions in blends of polyamides, polyesters and polycarbonate using copolymers. Macromol. Symp..

[18] Ellis T.S. (1997). Phase behaviour of polyamide-polyester blends: The influence of aromaticity. Polymer.

[19] Ridhore A., Jog J. (2013). Synergistic mechanical response of Nylon 6/Trogamid^®^ T blends. J. Appl. Polym. Sci..

[20] Yokozawa T., Asai T., Sugi R., Ishigooka S., Hiraoka S. (2000). Chain-growth polycondensation for nonbiological polyamides of defined architecture. J. Am. Chem. Soc..

[21] Yokozawa T., Ogawa M., Sekino A., Sugi R., Yokoyama A. (2002). Chain-growth polycondensation for well-defined aramide. Synthesis of unprecedented block copolymer containing aramide with low polydispersity. J. Am. Chem. Soc..

[22] Yokoyama A., Yokozawa T. (2007). Converting step-growth to chain-growth condensation polymerization. Macromolecules.

[23] Yokozawa T., Yokoyama A. (2009). Chain-growth condensation polymerization for the synthesis of well-defined condensation polymers and π-conjugated polymers. Chem. Rev..

[24] Masukawa T., Yokoyama A., Yokozawa T. (2009). Synthesis of well-defined polybenzamide-*block*-polystyrene by combination of chain-growth condensation polymerization and RAFT polymerization. Macromol. Rapid Commun..

[25] Ohishi T., Sugi R., Yokoyama A., Yokozawa T. (2008). Synthesis via chain-growth condensation polymerization and gelating properties of a variety of block copolymers-of *meta*- and *para*-substituted aromatic polyamides. Macromolecules.

[26] Huang C.F., Yokoyama A., Yokozawa T. (2010). Synthesis of polybenzamide-*b*-polystyrene block copolymer via combination of chain-growth condensation polymerization and atom transfer radical polymerization. J. Polym. Sci. Part A Polym. Chem..

[27] Huang C.-F., Ohta Y., Yokoyama A., Yokozawa T. (2011). Efficient low-temperature atom transfer radical coupling and its application to synthesis of well-defined symmetrical polybenzamides. Macromolecules.

[28] Yamazaki Y., Yokoyama A., Yokozawa T. (2012). Three-arm star block copolymers of aromatic polyether and polystyrene from chain-growth condensation polymerization, atom transfer radical polymerization, and click reaction. J. Polym. Sci. Part A Polym. Chem..

[29] Yoshino K., Yokoyama A., Yokozawa T. (2009). Well-defined star-shaped poly(*p*-benzamide) via chain-growth condensation polymerization: Use of tetra-functional porphyrin initiator to optimize star polymer formation. J. Polym. Sci. Part A Polym. Chem..

[30] Yoshino K., Yokoyama A., Yokozawa T. (2011). Synthesis of a variety of star-shaped polybenzamides via chain-growth condensation polymerization with tetrafunctional porphyrin initiator. J. Polym. Sci. Part A Polym. Chem..

[31] Ohishi T., Masukawa T., Fujii S., Yokoyama A., Yokozawa T. (2010). Synthesis of core cross-linked star polymers consisting of well-defined aromatic polyamide arms. Macromolecules.

[32] Sugi R., Hitaka Y., Yokoyama A., Yokozawa T. (2005). Well-defined star-shaped aromatic polyamides from chain-growth polymerization of phenyl 4-(alkylamino)benzoate with multifunctional initiators. Macromolecules.

[33] Yamazaki Y., Ajioka N., Yokoyama A., Yokozawa T. (2009). Synthesis of well-defined miktoarm star copolymers of aromatic polyether and polystyrene by chain-growth condensation polymerization and atom transfer radical polymerization. Macromolecules.

[34] Huang C.-F., Aimi J., Lai K.-Y. (2017). Synthesis of novel μ-star copolymers with poly(*N*-octyl benzamide) and poly(ε-caprolactone) miktoarms through chain-growth condensation polymerization, styrenics-assisted atom transfer radical coupling, and ring-opening polymerization. Macromol. Rapid Commun..

[35] Ohta Y., Fujii S., Yokoyama A., Furuyama T., Uchiyama M., Yokozawa T. (2009). Synthesis of well-defined hyperbranched polyamides by condensation polymerization of AB_2_ monomer through changed substituent effects. Angew. Chem. Int. Ed..

[36] Ohta Y., Kanou T., Yokoyama A., Yokozawa T. (2013). Synthesis of well-defined, amphiphilic poly(ethylene glycol)-*b*-hyperbranched polyamide. J. Polym. Sci. Part A Polym. Chem..

[37] Ohta Y., Kamijyo Y., Fujii S., Yokoyama A., Yokozawa T. (2011). Synthesis and properties of a variety of well-defined hyperbranched *N*-alkyl and *N*-H polyamides by chain-growth condensation polymerization of AB_2_ monomers. Macromolecules.

[38] Ohta Y., Kamijyo Y., Yokoyama A., Yokozawa T. (2012). Synthesis of well-defined, water-soluble hyperbranched polyamides by chain-growth condensation polymerization of AB_2_ monomer. Polymers.

[39] Harwood H.J., Ritchey W.M. (1964). The characterization of sequence distribution in copolymers. J. Polym. Sci. Part B Polym. Phys..

[40] Roman S.J., Madruga E.L., del Puerto M.A. (1983). Radical copolymerization of acrylic monomers. V. Copolymerization of *ortho*- and *para*-chlorophenyl acrylates with methyl methacrylate. J. Polym. Sci. Polym. Chem. Ed..

[41] Oswald H.J., Turi E.A., Harget P.J., Khanna Y.P. (1977). Development of a middle endotherm in DSC thermograms of thermally treated drawn pet yarns and its structural and mechanistic interpretation. J Macromol. Sci. Phys..

[42] Turi E.A. (1981). Analytical Sciences at Allied.

[43] Khanna Y.P., Kuhn W.P. (1997). Measurement of crystalline index in Nylons by DSC: Complexities and recommendations. J. Polym. Sci. Part B Polym. Phys..

