# Supplementary material for: Synthesis of Well-Defined Poly(N-H Benzamide-co-N-Octyl Benzamide)s and the Study of their Blends with Nylon 6
Source: Polymers (Basel). 2017 May 13;9(5):172. doi: 10.3390/polym9050172 (PMC6432416; doi:10.3390/polym9050172)
Supplement: Supplementary file 1 [file polymers-09-00172-s001.pdf]

Supplementary Materials: Synthesis of Well-Defined Poly(*N*-H Benzamide-*co*-*N*-Octyl Benzamide)s and the Study of their Blends with Nylon 6

Chih-Feng Huang, Miao-Jia Chen, Ching-Hsuan Lin and Yeo-Wan Chiang

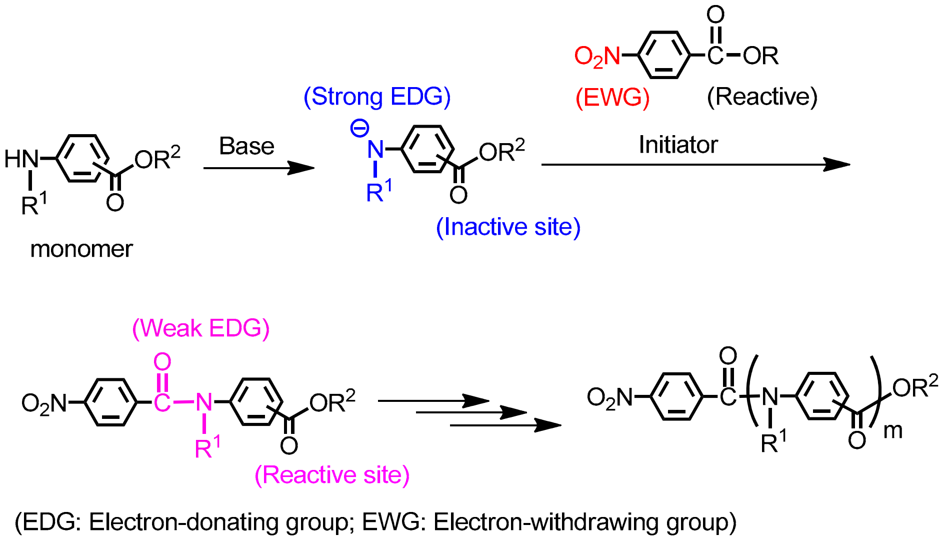

Scheme S1. Chain-growth condensation polymerization (CGCP) mechanism for the synthesis of well-defined.

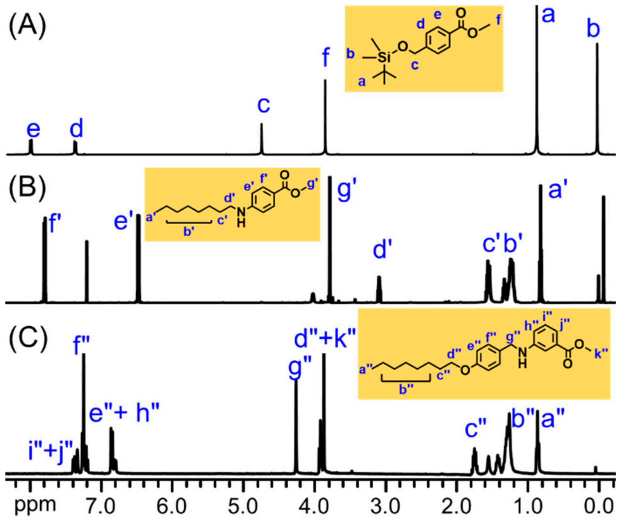

Figure S1. ^1H NMR spectra (400 MHz, $CDCl_3$) for the (**A**) initiator 4-(((*tert*-butyldimethyl silyl)oxy)methyl)benzoate, (**B**) monomer methyl 4-(octylamino)benzoate (M4OB) and (**C**) methyl 3-(4-(octyloxy)benzylamino)benzoate (M3OOB).

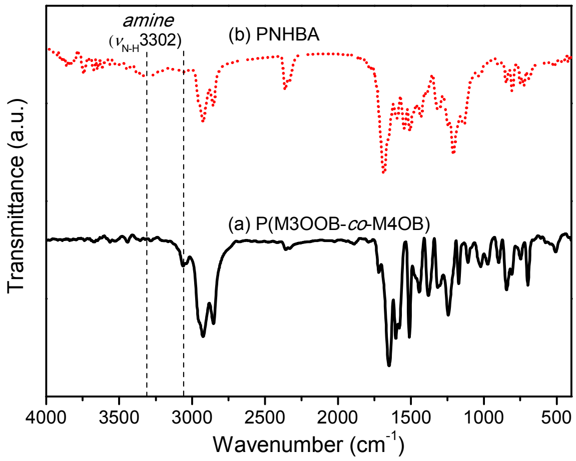

Figure S2. Representative FT-IR spectra of (**a**) P(M3OOB-*co*-M4OB) and (**b**) the resulting PNHBA after deprotection of the OOB group.

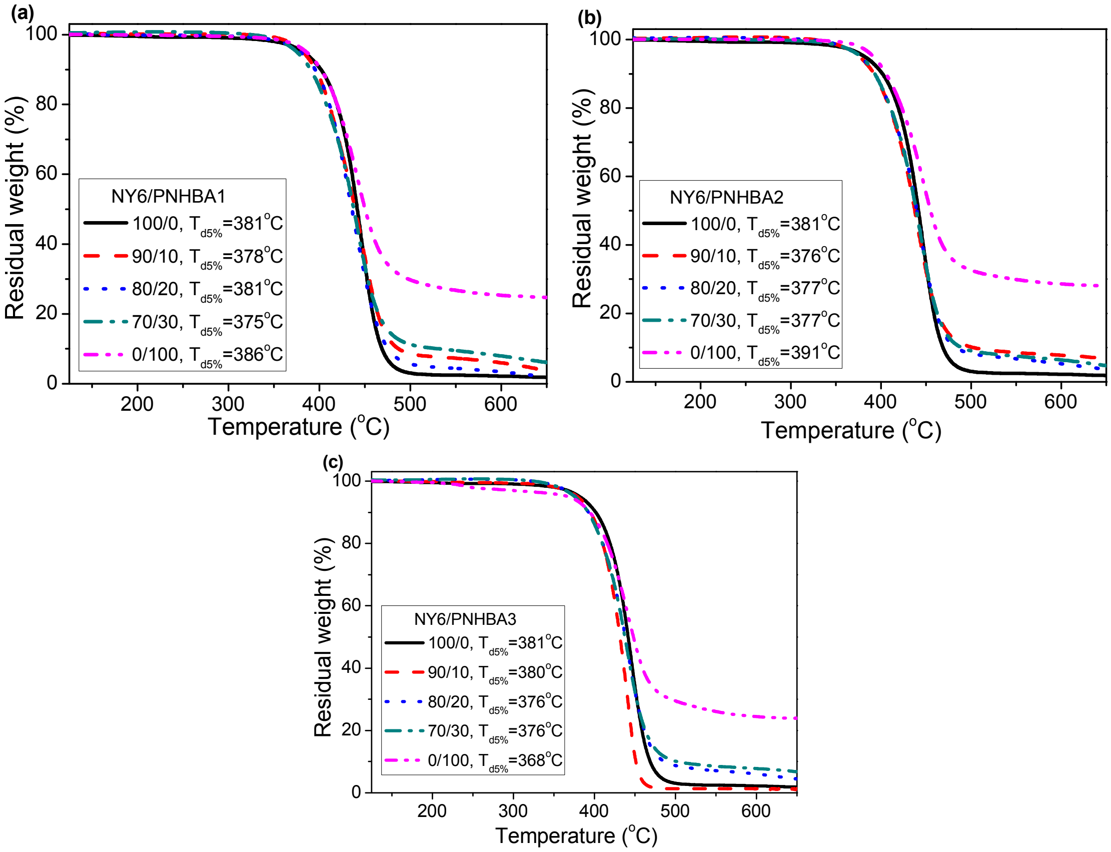

Figure S3. TGA traces and $T_{d5\%}$s of (**a**–**c**) NY6/PNHBA1–3 blends with different weight ratios.

Table S1. Characteristics of polybenzamide copolymers (PBAs).

Samples	Feeds Ratios (M3OOB/M4OB)	I_a/I_b $^{\alpha}$ (from ^{1}H NMR)	Unit Ratios in Copolymers (M3OOB/M4OB) $^{\beta}$
PBA1	90/10	1.00/0.11	90/10
PBA2	70/30	1.00/0.42	70/30
PBA3	50/50	1.00/0.88	48/52
PBA4	30/70	1.00/2.23	30/70
PBA5	10/90	1.00/9.49	10/90

$^{\alpha}$ I_a: intensity from 4.73 ppm and I_b: intensity from 3.79 ppm; $^{\beta}$ Estimated from the ratios of I_a/I_b corresponding to the resulting amounts of M3OOB/M4OB in copolymers.
